# Personalized treatment concepts in extraocular cancer

**DOI:** 10.1016/j.aopr.2024.02.003

**Published:** 2024-03-01

**Authors:** Sitong Ju, Alexander C. Rokohl, Yongwei Guo, Ke Yao, Wanlin Fan, Ludwig M. Heindl

**Affiliations:** a Department of Ophthalmology, University of Cologne, Faculty of Medicine and University Hospital Cologne, Kerpener Straße, Cologne, Germany; b Center for Integrated Oncology (CIO), Aachen-Bonn-Cologne-Duesseldorf, Cologne, Germany; c Eye Center, the Second Affiliated Hospital of Zhejiang University School of Medicine, Hangzhou, China

**Keywords:** Periocular malignancy, Eyelid, Conjunctiva tumor, Immunotherapy, Neoadjuvant therapy

## Abstract

**Background:**

The periocular skin is neoplasms-prone to various benign and malignant. Periocular malignancies are more aggressive and challenging to cure and repair than those in other skin areas. In recent decades, immunotherapy has significantly advanced oncology, allowing the autoimmune system to target and destroy malignant cells. Skin malignancies, especially periocular tumors, are particularly sensitive to immunotherapy. This technique has dramatically impacted the successful treatment of challenging tumors.

**Main text:**

Extraocular cancers, including eyelid (basal cell carcinoma, squamous cell carcinoma, melanoma, merkel cell carcinoma), conjunctival tumors (conjunctival melanoma, ocular surface squamous neoplasia) and other rare tumors, are unique and challenging clinical situations. Several genetic alterations associated with the pathogenesis of these diseases have been identified, and molecular mechanism are essential for the development of the immunotherapy agents, such as Hedgehog pathway inhibitors (vismodegib and sonidegib) for basal cell carcinoma, BRAF/MEK inhibitors (vemurafenib, dabrafenib, and encorafenib) for melanoma, and immune checkpoint inhibitors (Avelumab, pembrolizumab) for Merkel cell carcinoma.

**Conclusions:**

The optimal treatment for periocular skin cancer depends on the type and size of the tumor and whether it involves orbital and adnexal structures. Adjuvant and neoadjuvant therapy with chemotherapy-targeted therapies and immune checkpoint inhibitors should be considered based on tumor type, tumor molecular profile, expected response rate, and candidacy for systemic treatment.

## 1 Introduction

Skin cancer frequently occurs in the periocular region due to prolonged exposure of the head and neck to ultraviolet light.^1^ Non-metastatic skin cancers are usually treated by radical excision or radical radiotherapy. However, these techniques have significant morbidity and quality of life implications in the periocular region, especially for large tumors, and may be rejected by some patients. Extraocular cancers, including eyelid (basal cell carcinoma, BCC; squamous cell carcinoma, SCC; eyelid melanoma, EM; Merkel cell carcinoma, MCC) and conjunctival tumors (conjunctival melanoma,CM; ocular surface squamous neoplasia, OSSN), are unique and challenging clinical situations. Effective radical systemic therapies for these cancers are very limited and treatment outcomes are unclear. Some periocular skin cancers invading the orbit may require orbital resection, which can severely affect vision and appearance and have a significant impact on quality of life. In contrast, recent years have seen advances in the study of the molecular pathways that drive cancer development and the role of the immune system in cancer surveillance, as well as the emergence of immunotherapy and targeted therapies for locally advanced and metastatic skin cancers.^2^, ^3^, ^4^, ^5^ These advances have prompted research into neoadjuvant systemic therapies to minimize definitive treatment morbidity and improve long-term outcomes. Recent clinical studies have begun to provide an evidence base for using neoadjuvant targeted and immunotherapies in treating skin cancer.^6^ They may soon lead to changes in treatment guidelines for these malignancies.

## 2 Literature search

**Fig. 1:**
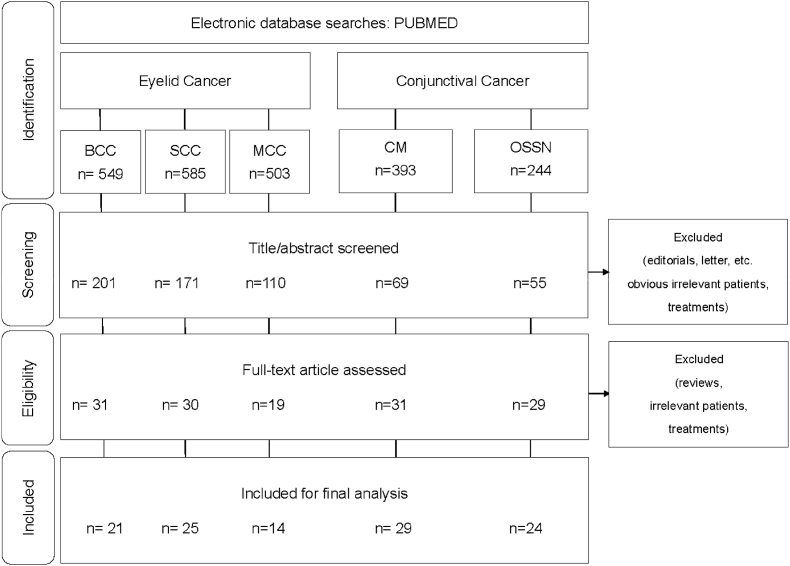
Systematic review flow diagram. BCC, Basal Cell Carcinoma. SCC, Squamous Cell Carcinoma. MCC, Merkel Cell Carcinoma.CM, Conjunctival Melanoma. OSSN, Ocular Surface Squamous Neoplasia.

The PubMed database was searched with no specific filters for an overview of the topic. A literature search was limited to the articles published in the last ten years (until December 2023) for the summary and the critical review of the recent advancements (Fig. 1).

For the case of eyelid cancer, scientific terms such as "basal cell carcinoma" or "BCC" in combination with "Hedgehog pathway" or "Hedgehog pathway inhibitors" or "immune checkpoint inhibitors" or "immunotherapy" or "Vismodegib" or "Sonidegib", "biomarkers", "cancer", "tumors"; "eyelid melanoma"; "squamous cell carcinoma" or "SCC" combined with "immune checkpoint inhibitors" or "immunotherapy"; "Merkel cell carcinoma" or "MCC" or "Merkel cell polyomavirus" or "MCPyV" combined with "Programmed Death-Ligand 1" or "PD-L1" or "immunotherapy" or "immune checkpoint inhibitors" or "Avelumab" or "pembrolizumab" were searched.

In the case of conjunctival cancer, keywords such as "conjunctival melanoma" or "CM" or "ocular surface squamous neoplasia" or "OSSN" in combination with "MAPK pathway" or "BRAF inhibitors" or "MEK inhibitors" or "CTLA-4 inhibitor" or "PD-1 inhibitor" or "immunotherapy" or "vemurafenib" or "dabrafenib" or "encorafenib" or "trametinib" or "binimetinib" or "cobimetinib" or "ipilimumab" were used.

## 3 Personalized treatment in eyelid cancer

### 3.1 Personalized treatment in eyelid basal cell carcinoma

Basal cell carcinoma (BCC) is the most common skin cancer, especially in the white population, comprising 75% of all^7^ According to some studies, higher prevalence was found on the sun-exposed face, head, and neck, and 40–74% of BCCs were located in the periocular area.^8^^,^^9^ Sporadic BCC is associated with a variety of factors, including UV (ultraviolet) exposure (sunlight or indoor tanning), old age, lightly pigmented skin and hair, family history of BCC, immunosuppression (solid organ transplantation), and genetic factors.^10^, ^11^, ^12^ Since most BCCs have indolent bio-behavioral characteristics and are easily detected at an early stage, it can be cured by local surgical excision or topical therapy (e.g., imiquimod and fluorouracil). In some cases, such as advanced BCC, it can be metastatic. Locally infiltrating into adjacent and deeper structures, including regional lymph nodes, lungs, spine, bone marrow, and pelvic bone, leads to extensive tissue destruction.^13^

**Fig. 2:**
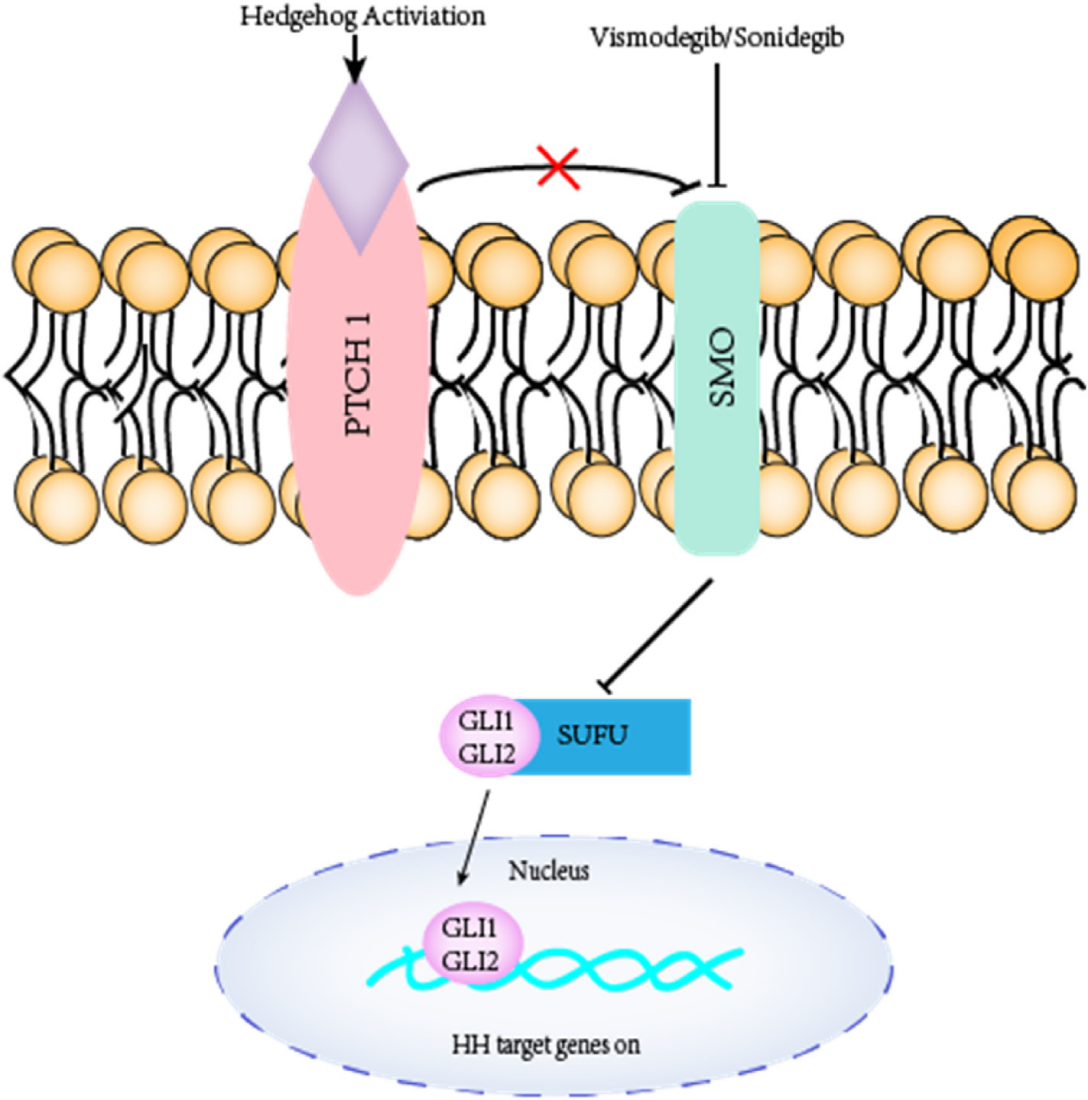
Hedgehog signaling pathway PTCH1 inhibits SMO in the absence of HH signaling ligand; when ligand is present, it binds to the transmembrane protein PTCH1, and the membrane spanning receptor SMO rescinds the PTCH1-mediated inhibition, leading to activation of the Gli transcription factor and entry into the nucleus to stimulate cell division and tumorigenesis. Several HPIs, such as vismodegib and sonidegib, bind SMO and inhibit reactivation of the HH pathway, available for cancer therapy. PTCH1, Patched-1; SMO, Smoothened; HH, Hedgehog; HPIs, hedgehog pathway inhibitors.

Several genetic alterations associated with the pathogenesis of BCC have been identified these years, including melanocortin 1 receptor gene (*MC1R*), oculocutaneous albinism type 2 gene (*OCA2*), *p53*, agouti signaling protein (*ASIP*) and tyrosinase (*TYR*).^7^^,^^14^^,^^15^ Among all genetic factors, the hedgehog (HH) signaling pathway would aberrantly activated by Patched (*PTCH1* and *PTCH2*) loss-of-function somatic mutations and the G-protein coupled receptor smoothened (*SMO*) activating mutations (Fig. 2), which have been founded in almost all BCCs.^16^, ^17^, ^18^ This molecular mechanism is essential for hedgehog pathway inhibitors (HPIs) development to treat advanced BCC [locally-advanced BCC (laBCC) and metastatic BCC (mBCC)] systemically.^19^ HPIs, such as Vismodegib and Sonidegib, inhibit the activation of HH target genes by binding to SMO and selectively and effectively inhibiting the HH pathway.^20^, ^21^, ^22^

#### 3.1.1 Vismodegib

Vismodegib (150 ​mg/day) is a first-in-class, oral small molecule, HH pathway inhibitor (HPI) that was approved in 2012 by the FDA in the US and 2013 by EMA in the EU for the treatment of adults with laBCC inappropriate for surgery or radiotherapy, or with symptomatic mBCC.^23^ EAS(expanded access study), is the largest multicenter, two-cohort, open-label study of Vismodegib in advanced BCC prior to SafeTy Events in VIsmodEgib (STEVIE).^2^ In total, 119 patients were enrolled, and 95 (laBCC n ​= ​56, mBCC n ​= ​39) were efficacy-evaluable. Overall response rate (ORR) achieved by patients with laBCC was 46.4% (CR 10.7%, PR 35.7%), and mBCC was 30.8% (CR 5.1%, PR 25.6%). 47 patients (49.5% of all) experienced stable disease (laBCC n ​= ​27, mBCC n ​= ​20). Totally, 94.6% of patients in the laBCC group and 82.1% in the mBCC group achieved complete response(CR), partial response(PR), or stable disease.^2^

Safety and encouraging response rates of vismodegib were found in phase II STEVIE trial,^24^ with 1161 patients and a 17.9-month median follow-up time. Response rates were 68.5% in patients with laBCC and 36.9% in mBCC, and patients with Gorlin syndrome responded better to vismodegib (81.7% in laBCC and 80.0% in mBCC).^24^

Based on ERIVANCE BCC, a phase II, multicenter, international, dual-cohort, nonrandomized study, 33 mBCC patients and 63 inoperable laBCC patients received 150 ​mg of vismodegib daily. With a 7.6-month median response duration, the ORR was 30.3% in patients with mBCC(10 ​PR) and 42.9% in laBCC. In the laBCC cohort of 63 evaluable patients, thirteen (20.6%) and fourteen (22.2%) achieved CR and PR, respectively.^3^ The final 39-month update showed ORR was 48.5% (mBCC) and 60.3% (laBCC). Furthermore, the therapy was clinically efficacious against aggressive histologic subtypes, with an ORR of 53.8% in laBCC and 85.7% in mBCC.^25^

VISMONEO (Vismodegib Neoadjuvant) study published in 2021, the primary endpoint defined as downstaging of the surgical procedure in 10 month treatment period, enrolled 55 patients with laBCC(19 patients with periocular laBCC).^26^ 44 patients (80%) presented with downstaging after vismodegib treatment, and 27 of them had a complete clinical response. A 71% ORR rate by Response Evaluation Criteria in Solid Tumors v1.1(RECIST) and 66% mean tumor size reduction were achieved in 6-months mean treatment duration. The recurrence rate was as high as 36.4% (16/44) who responded to vismodegib treatment during a 3-year follow-up, which may due to the fact that a larger proportion of patients did not receive surgical treatment ultimately.

The VISORB (Vismodegib for Orbital and Periocular Basal Cell Carcinoma) Trial^27^ enrolled 34 patients with orbital and extensive periocular BCC (opBCC), and 150 ​mg vismodegib was taken per day orally for up to 12 months. A novel Visual Assessment Weighted Score (VAWS) consisting of 8 items, such as preservation of visual organs, acuity, extraocular motility, and lacrimal drainage, was defined as the primary endpoint in addition to standard ophthalmic exams. all patients maintained successful VAWS outcomes. The most significant tumor size reduction was seen after six months of treatment, averaging 22% of baseline size, as measured by physical examination. Twenty-seven patients (79.4%) choose to take excision before the endpoint, and 67%^18^ showed complete histologic clearance. Nineteen patients (56%) demonstrated CR by physical examination, and 47%^16^ by MRI/CT.^27^ These findings provide convincing evidence for vismodegib as a neoadjuvant to surgery or in combination with other therapies to improve visual function and achieve an actual cure.

#### 3.1.2 Sonidegib

Similarly, in 2015, Sonidegib (200 ​mg/day) was authorized for the treatment of adults with recurrence laBCC following surgery or radiation or those who are not amenable to radiotherapy or curative surgery.

A phase II randomized, double-blind BOLT study [Basal Cell Carcinoma Outcomes with LDE225 (sonidegib) Treatment],^28^ 230 patients in total were enrolled in this trial randomized to different dose groups: 194 patients with laBCC (66, 200-mg; 128, 800-mg) and thirty-six with mBCC (13, 200-mg; 23, 800-mg). Sonidegib Treatment continued for up to 42 months, and the primary endpoint was the ORR evaluated with RECIST v1.1. The ORRs for laBCC and mBCC were 48% and 41.7% for the 200-mg and 800-mg groups, respectively. laBCC patients achieved higher ORRs with 56%(200-mg) and 46.1% (800-mg) than mBCC patients [8% (200 ​mg) and 17% (800 ​mg)]. Moreover, the disease control rate (DCR) for the 200-mg dose exceeded 90% both in patients with laBCC(91%) and mBCC (92%); for the 800-mg dose, the DCR was 82.0% in laBCC and 91% in mBCC.^28^ No additional efficacy was found from different dose groups. The results support sonidegib as a viable long-term treatment option for advanced BCC patients.

### 3.2 Personalized treatment in eyelid squamous cell carcinoma

Squamous cell carcinoma (SCC) accounts for 5%–10% of all eyelid skin cancers, and caused by the malignant proliferation of keratinocytes derived from the epidermis and adnexal structures.^29^ The prevalence is higher in males, lower eyelid (inner canthus), fair-skinned/hair, and area with high UV exposure.^30^ SCC usually presents as a series of progressive malignancies, with an early stage of precancerous actinic keratosis (AK), then advancing to SCC in situ (SCCIS), invasive SCC, and finally, metastatic SCC (mSCC). Several genetic mutations (e.g. *TP53*, *CDKN2A*, *NOTCH1* and *NOTCH2*, *EGFR* and *TERT*) and molecular pathways (*RAS-RAF-MEK-ERK* and *PI3K-AKT-mTOR*), epigenetic modifications, viral tumorigenesis, and micro-environmental changes are involved in this process. Surgery remains the mainstay of treatment, supplemented by appropriate therapies (topical cryotherapy/radiotherapy/chemotherapy, systemic therapy), depending on the location of the lesion, the tumor cell remains in the margins and the existence of metastases.

#### 3.2.1 Topical therapy

5-fluorouracil (5-FU), a structural analogue of thymine that inhibits thymidylate synthase, causing DNA synthesis inhibition and ultimately suppressing the rapid proliferation of cancer cells.^31^ Interferon alpha (INFα) is a naturally occurring low molecular weight glycoprotein with anti-tumor properties, including apoptosis induction, angiogenesis inhibition and cancer cell cycle time prolongation.^32^ Mitomycin-C (MMC) is an agent with anti-tumor properties isolated from Streptomyces caespitosus, which undergoes metabolic activation under aerobic conditions which subsequently leads to DNA strand breaks, impaired DNA synthesis and a cytotoxic environment, ultimately inducing apoptosis.^33^^,^^34^ For cases with positive margins due to inadequate surgical resection, extensive tumor, high recurrence rate or deep local infiltration, topical postoperative treatment with MMC, 5FU or INFα may be used to achieve better outcomes.^35^, ^36^, ^37^, ^38^

#### 3.2.2 Targeted therapy

Epidermal growth factor receptor (EGFR), a transmembrane tyrosine kinase receptor of the ErbB family, is commonly overexpressed in SCC, making it a potential target for targeted therapies in locally advanced SCC(laSCC).^39^ All cases had moderate to strong EGFR expression, as shown in a retrospective study of five conjunctival SCC cases.^40^ Cetuximab, an anti-EGFR antibody, has been proved to be effective in head and neck SCC in several clinical trials.^41^, ^42^, ^43^ For overall cutaneous SCC, a phase II single-agent trial with 36 cutaneous SCC patient (five with primary head and neck SCC), 25 patients (69%) achieved disease control. However, no studies have shown that anti-EGFR antibody therapy are effective in patients with eyelid SCC.

#### 3.2.3 Immune checkpoint inhibitors

In 2018, the PD-1 blocking antibody cemiplimab became the first medicine to receive FDA approval for the treatment of patients with aSCC.^44^ In a phase 1 expansion cohort study of laSCC or mSCC patients, and pivotal phase 2 metastatic-disease cohort study of mSCC patients, OR was achieved in 46.7% of 75 patients with mSCC and in 48.5% of 33 patients with laSCC. In addition, 60% of patients with mSCC and 63% with laSCC remained PR/CR for over 6 months.^45^

### 3.3 Personalized treatment in eyelid melanoma

Primary eyelid melanoma (EM) is thought to arise from lentigo maligna, a form of melanoma in situ (MIS). It is an extremely rare disease with an incidence of less than 1%,^46^ and thus its etiological factors (UV) are mostly deduced based on the experience of facial skin melanomas.^47^ The treatment of EM is essentially surgical excision combined with eyelid reconstruction.^48^ Although immunotherapy has been widely used in the melanoma treatment, the therapeutic effect in EM is still unclear.^49^

### 3.4 Personalized treatment in eyelid merkel cell carcinoma

Merkel cell carcinoma (MCC) is a rare, life-threatening non-melanoma skin cancer that originates from Merkel cells in the epidermis Merkel cell carcinoma (MCC) is a non-melanoma skin cancer originating from epidermal Merkel cells.^50^ It is a highly aggressive and rare skin cancer with unique neuroendocrine characteristics, early metastasis, high morbidity, poor prognosis after metastasis, and can be life-threatening. Old age, ultraviolet light exposure, immunocompromised status, and exposure to human polyomavirus are associated with the pathogenesis of the disease.^51^^,^^52^

Majority of MCC cases are related to Merkel cell polyomavirus (MCPyV) infection. PD-L1, a transmembrane protein that is aberrantly expressed in a variety of neoplastic cells or tumor stromal immune cells,^53^ is observed in both MCPyV+ and MCPyV- MCC patients, and more common in MCPyV ​+ ​cases.^54^ Higher overall survival (OS) and progression-free survival (PFS) have been reported in several studies in patients with PD-L1+ tumors.^54^^,^^55^ Previous findings supported that PD-L1 signaling pathway blocking can be a possible direction for MCC immunotherapy, and several immune checkpoint inhibitors (ICIs) have been approved for the treatment of advanced MCC in recent years.

#### 3.4.1 Avelumab

Avelumab, a anti-PD-L1 antibody, was approved in 2017 by the FDA and EMA for metastatic MCC (mMCC) treatment.^51^ JAVELIN Merkel 200, a cohort of this single-arm phase II trial enrolled 88 patients with mMCC or disease progression after prior chemotherapy, published in 2018(56). All patients received avelumab 10 ​mg/kg intravenously every two weeks. The ORR was 33.0%, and 11.4% (10 patients) were observed to have a CR with a 40.5-month median response duration. Moreover, 81.8% of long-term survivors (OS ​>36 months) had PD-L1+ tumors.^56^ Similarly, another study with 116 mMCC patients receiving the same treatment was reported in 2021(57). ORR was 39.7%, and the median OS was 20.3 months during follow-up. Thirty-five patients had a 6-month or longer response, giving a durable response rate of 30.2%. Moreover, patients with PD-L1+ and MCPyV- tumors had higher response rates.^57^ These results suggest that avelumab could be an efficient treatment for MCC patients, especially those with PD-L1+ tumors, who likely have an increased probability of long-term OS.

A multicenter chart review study in the USA investigated real-world(rw) clinical outcomes in advanced MCC (aMCC) patients.^58^ Ninety patients with aMCC (18%, stage IIIB; 82%, stage IV) received avelumab. With a 20.8-month median follow-up duration, 81% (73/90) patients received avelumab as first-line treatment (rwORR 75.3%, OS 41.7 months), while 19% (17/90) received a second-line or later treatment (rwORR 64.7%, OS 15.9 months). Compared with patients treated with second-line therapy, patients who received avelumab as a first-line treatment tended to have a better prognosis. Avelumab-treated patients had a rwORR of 73% and a median OS of 30.7 months. Results from this study were consistent with other real-world studies and those observed during clinical trials.

Meanwhile, several real-world studies also illustrated that the efficacy of avelumab is not appear to be limited even if the MCC patient is immunocompromised. A study by Walker et al.^59^ reported that the ORR of immunocompromised was 37.5%, similar to the overall cohort (46.7%). And response duration was also similar in both groups. According to Averbach et al. study,^60^ 22.6% of the total 62 patients were immunocompromised, and no differences were revealed in outcomes between the immunocompromised (ORR,61%) and immunocompetent patients (ORR,53.8%), and ORR 59.0% in all.

Although to date there have been no clinical trials evaluating the efficacy of avelumab specifically in primary or metastatic cases of eyelid or periocular MCC, the previous studies provide a solid basis for future investigation.

#### 3.4.2 Pembrolizumab

In 2018, the FDA granted accelerated approval to another anti-PD-1 antibody, pembrolizumab, for adult and pediatric patients with recurrent locally aMCC or mMCC.^61^ Based on a multicenter, non-randomized, open-label trial-CITN-09(Cancer Immunotherapy Trials Network protocol 9),^62^ 26 patients with recurrent locally aMCC or mMCC without receiving prior systemic therapy received pembrolizumab 2 ​mg/kg every 3 weeks and 25 patients assessed by RECIST 1.1. With a median follow-up of 33 weeks, the ORR was 56% (16% CR, 40% PR). The median response duration was not reached. A total of 17 patients (65%) had MCPyV ​+ ​tumors and the response rate was 62%, while 44% among those with MCPyV- tumors.

In 2019, a two-year-long follow-up study validated these early findings with encouraging long-term outcomes.^63^ With a 14.9 months median follow-up time of 50 patients, ORR was 56% (24% CR, 32% PR), with ORRs of 59% in MCPyV+ and 53% in MCPyV- tumors. Among 28 responding patients, the 2-year OS rate was 68.7%.

Pembrolizumab demonstrated durable tumor control and a promising OS compared to first-line chemotherapy.

## 4 Personalized treatment in conjunctival cancer

### 4.1 Personalized treatment in conjunctival melanoma

Conjunctival melanoma (CM) is a rare, aggressive, and potentially fatal malignant neoplasm originating from melanocytes in the basal layer of the conjunctival epithelium.^64^ The incidence of CM in the white population is 0.2–0.8 cases/million.^65^ It has slowly increased over the last few decades, possibly due to UV exposure and the increasing aging population. Other risk factors include light skin color, indoor tanning, multiple moles, family history, inherited cancer syndrome, and immunosuppression.^64^

CMs occur more frequently in bulbar conjunctiva and other areas exposed to sunlight.^66^ Originate mainly from melanocytic precursor lesions, like primary acquired melanosis with atypia (up to 75%) or a pre-existing nevus (less than 10%), whereas *de novo* cases account for 15%–25% of CMs.^66^^,^^67^ Localized lesion is generally treated by surgical excision and adjuvant therapy (cryotherapy, radiotherapy, or topical chemotherapy), while extensive lesions on the palpebral conjunctiva or ocular surface may require wider treatment (orbital exenteration).^64^^,^^68^^,^^69^

#### 4.1.1 BRAF and MEK inhibitor

*BRAF* mutations have been found in about one-third of CMs,^70^, ^71^, ^72^, ^73^ and nearly all occur at codon 600 (V600).^74^, ^75^, ^76^ Constitutive activation of BRAF proteins results from these oncogenic mutations, and promotes tumor growth via constitutive downstream activation of the MAPK pathway (MEK1/2 and ERK1/2).^73^^,^^77^^,^^78^ Drugs targeting BRAF and MEK can inhibit the MAPK pathway, and several BRAF inhibitors (vemurafenib, 2011; dabrafenib, 2013; encorafenib, 2018) and MEK inhibitors (trametinib, 2013; binimetinib, 2018; cobimetinib, 2020) have been approved for cutaneous melanoma treatment since 2011. Similarly, BRAF inhibitors are also the most well established CM-targeted therapy medication type.

#### 4.1.2 Other inhibitors

Dendritic cells (DCs) are critical in triggering strong immune responses or inducing tolerance, and these processes are strictly regulated. Normally, T cells are suppressed or differentiate into regulatory T cells when dendritic cells express PD-1 or cytotoxic T-lymphocyte-associated protein 4 (CTLA-4).^79^ Tumor cells imitate this behavior of DCs by expressing CTLA-4 (CD80 and CD86) or PD-1 (PDL-1), effectively inhibiting cytotoxic T-cell activation and inducing tumor cells to evade recognition and clearance by the immune system, thus to create an immune-tolerant environment. Immune checkpoint inhibitors (e.g. ipilimumab, nivolumab and pembrolizumab) are monoclonal antibodies that target the ligands of these inhibitory T-cell receptors.^80^ This inhibition is blocked by binding to regulatory receptors on T cells, thereby activating the immune system against tumor cells.^81^ Both the PD-1(nivolumab, pembrolizumab) and CTLA-4 inhibitor (ipilimumab) are approved for the treatment of metastatic cutaneous melanoma, and they are generally used alone and in combination when necessary.^82^^,^^83^

Until December 2023, some CM cases reported of treated with BRAF/MEK inhibitor, PD-1 or CTLA-4 inhibitor were found (Table 1).

**Table 1:** CM targeted therapy case reports overview.

Study	Patient（Gender, age/year）	Type of CM	Type and Dosage Immunotherapy	Other treatments	Clinical outcome
**primary CM**					
Pahlitzsch M, et al. (2014)^4^	F, 80y	Recurrence. Metastases (−) *BRAF* mutation (exon 15)	vemurafenib, 16 months	Incomplete excision, Plaque brachytherapy, Resection(later)	Good response, tumor decrease, stable for 3y. Then deterioration general health.
Kim et al.(2020)^108^	M, 52y	Recurrence, metastases (−). *BRAF* mutation (V600)	dabrafenib 150 ​mg ​b.i.d.+ trametinib 2 ​mg q.d.	–	Good response, complete resolution, 15 months, alive, no metastases
**Metastatic CM**					
Weber JL, et al. (2013)^109^	M, 45y	Metastatic (subcutaneous, lung, bone) *BRAF* muta (v600e).	vemurafenib 960 ​mg ​b.i.d. 14month	CM: resection	good response, progression after 2 months.
Griewank KG, et al. (2013)^110^	M, 43y	Metastatic CM (intramuscular, lung, brain) *BRAF* mutation not reported.	dabrafenib	CM: resection, Ruth, partial irradiation. Mets: dacarbazine	PR. 62% tumor reduction. New lesions after 6 months.
Maleka A, et al. (2016)^111^	F, 53y	Metastatic (orbit, parotid gland, lung, brain) *BRAF* mutation (v600e)	vemurafenib 960 ​mg ​b.i.d., later 720 ​mg ​b.i.d. (skin rash).	CM: resection, cryotherapy, mytomicin-c, enucleation. Mets: Temozolomide, AdCD40L with cyclophosphamide, Brain radiotherapy.	good response, reduction of metastases. After 4 months, metastases re-appearance. Death.
Pinto Torres S, et al. (2017)^112^	F, 59y	Metastatic (Oropharyngeal wall) *BRAF* mutation (v600e)	vemurafenib 960 ​mg ​b.i.d.; later 480 ​mg ​b.i.d.(arthralgia, diarrhea, skin rash), 1 month	CM: excision, Mets: radiotherapy 20Gy/5 fractions	CR. No recurrence in 34 months. Developed breast cancer.
**Combined therapy**					
Dagi Glass LR et al. (2017)^113^	F, 61y	Recurrent, metastases (lymph) *BRAF* mutation (v600e)	1.dabrafenib ​+ ​trametinib; 2. vemurafenib (nausea, vomiting); 3. pembrolizumab (progression); 4.vemurafenib ​+ ​cobimetinib (progression)	CM: excision, cryotherapy. Lymph metastases: parotidectomy ​+ ​neck dissection	1: good for 1.5 months, then nausea and vomiting. 2-3: mixed, not complete. 4: eventually good response.
Rossi E, et al. (2019)^114^	M, 70y	Metastatic (lymph) *BRAF* mutation (v600e)	dabrafenib 150 ​mg ​b.i.d. ​+ ​trametinib 2 ​mg q.d.	CM: excision lymph metastases: parotidectomy ​+ ​neck dissection	Good response, Alive 1y later.
**Other genetic mutations**					
Francis JH et al.(2023)^115^	F, 50y	*NRAS Q61R* mutation	ipilimumab 1 ​mg/kg ​+ ​nivolumab 3 ​mg/kg, nivolumab 480 ​mg maintenance. 4 months	stereotactic radiosurgery 30 ​Gy/5 fractions	Good response, 11months, vision improve
Fan K et al. (2023)^116^	F	Recurrent, metastases (nose, lymph) *b-raf and v-raf murine sarcoma viral oncogene homolog B1-negative*	ipilimumab ​+ ​nivolumab, nivolumab maintenance, 4 cycles	CM:complete resection	CR, No recurrence,1y

**Abbreviations:** CM, conjunctival melanoma; PR, partial response; CR, complete response; M, male; F, female.

∗ This table modified from Niels J et al.^117^

### 4.2 Personalized treatment in ocular surface squamous neoplasia

Ocular surface squamous cell neoplasms (OSSN) are abnormal growths of hypoplastic squamous epithelial cells on the ocular surface, including conjunctival intraepithelial neoplasia (CIN), cornea neoplastic lesions, and SCC.^84^ The main associated risk factors are UV radiation, immunosuppression/HIV, human papilloma virus (HPV), and oncogene p53 mutation or deletion.^85^^,^^86^

While surgical resection is still the preferred method for small lesions, topical therapies such as 5-FU,^87^, ^88^, ^89^ INFα-2b,^89^, ^90^, ^91^, ^92^ MMC,^93^, ^94^, ^95^, ^96^ and immunotherapy PD-1 checkpoint inhibitors gradually becoming an essential treatment modality to improve outcomes.

#### 4.2.1 5-FU

5-FU became the primary and surgical adjuvant for OSSN since the 1980s.^31^ Multiple studies supported the efficacy of 5-FU as primary treatment, 82%–100% of patients achieved CR with a recurrence rate of less than 11% at one year.^88^^,^^89^^,^^97^

#### 4.2.2 IFNα-2b

IFNα-2b has been used as an effective alternative to surgical removal, either by subconjunctival injection, eye drops, or both, as a primary or adjunctive treatment modality for OSSN.^98^^,^^99^ In a study enrolling 18 cases with giant OSSN, 12 eyes received topical IFNα-2b, 3 with injections, other three with combination of both. In 72% (13 cases) of all achieved complete control and 28% (5 cases) were reduced in size.^100^

#### 4.2.3 MMC

MMC can be used as a primary therapeutic agent, as an intraoperative adjunct to surgical resection, or as a post-operative agent in margin-positive patients.^96^^,^^101^ Studies have illustrated that more than 80% of cases achieved CR or PR, providing convincing evidence of the efficacy of topical MMC therapy.^96^^,^^101^^,^^102^

#### 4.2.4 PD-1 inhibitors

Relevant studies have demonstrated that the PD-L1 expression level in OSSN tumor cells correlates with the invasion level.^103^^,^^104^ The FDA has approved the anti-PD-1 drugs pembrolizumab and cemiplimab for skin SCC treatment.^105^ According to a study from Demirci H et al.,^106^ five patients with advanced conjunctival SCC were treated with systemic PD-1 inhibitors and four achieved CR without recurrence between 2 and 11 months. Similarly, patients with recurrent SCC continued to progress after repeated radiation treatments. Cemiplimab was then delivered intravenously (3 ​mg/kg every 2 weeks), and no disease progression was demonstrated on radiological examination after the 19-month continuous treatment.^107^

## 5 Conclusions

The optimal treatment for periocular skin cancer depends on the type and size of the tumor and whether it involves orbital and adnexal structures. Destruction of visual structures and facial appearance cannot be avoided in most patients undergoing radical surgery or radiation therapy. Neoadjuvant therapy with chemotherapy-targeted therapies and immune checkpoint inhibitors should be considered based on tumor type, tumor molecular profile, expected response rate, and candidacy for systemic therapy. Before the surgery, the risks of the treatment process and medication toxicities should be fully understood by patients. Complex cases should involve multidisciplinary consideration of oculofacial surgeons, radiation, and medical oncologists, depending on the patient's preferences and goals.

## Study approval

Not Applicable.

## Author contributions

The authors confirm contribution to the paper as follows: Conception and design of study: LH, KY; Drafting the manuscript: SJ, WF; Figures and Tables set up: SJ, WF; Revision of Manuscript: WF, AR. All authors reviewed the results and approved the final version of the manuscript.

## Funding

This work was supported by the 10.13039/501100004543State Scholarship Fund from China Scholarship Council (No.202108080189).

## Editorship disclosure

Given their role as Editor-in-Chief, Ke Yao had no involvement in the peer-review of this article and has no access to information regarding its peer-review. Full responsibility for the editorial process for this article was delegated to Andrzej Grzybowski, MD.

## Declaration of competing interest

The authors declare that they have no known competing financial interests or personal relationships that could have appeared to influence the work reported in this paper.
